# Open questions in the social lives of viruses

**DOI:** 10.1111/jeb.14203

**Published:** 2023-11

**Authors:** Asher Leeks, Lisa M. Bono, Elizabeth A. Ampolini, Lucas S. Souza, Thomas Höfler, Courtney L. Mattson, Anna E. Dye, Samuel L. Díaz-Muñoz

**Affiliations:** 1 Department of Ecology and Evolutionary Biology, Yale University, New Haven, Connecticut, USA; 2 Quantitative Biology Institute, Yale University, New Haven, Connecticut, USA; 3 Department of Biological Sciences, Texas Tech University, Lubbock, Texas, USA; 4 Department of Biochemistry & Molecular Biology, Medical University of South Carolina, Charleston, South Carolina, USA; 5 Department of Ecology & Evolutionary Biology, University of Tennessee, Knoxville, Tennessee, USA; 6 Institute of Virology, Freie Universität Berlin, Berlin, Germany; 7 Department of Microbiology and Molecular Genetics, University of California Davis, Davis, California, USA; 8 Department of Plant and Microbial Biology, North Carolina State University, Raleigh, North Carolina, USA; 9 Genome Center, University of California Davis, Davis, California, USA

**Keywords:** cheating, conflict, cooperation, evolutionary theory, natural history, population genetics, social evolution, virology, virus evolution

## Abstract

Social interactions among viruses occur whenever multiple viral genomes infect the same cells, hosts, or populations of hosts. Viral social interactions range from cooperation to conflict, occur throughout the viral world, and affect every stage of the viral lifecycle. The ubiquity of these social interactions means that they can determine the population dynamics, evolutionary trajectory, and clinical progression of viral infections. At the same time, social interactions in viruses raise new questions for evolutionary theory, providing opportunities to test and extend existing frameworks within social evolution. Many opportunities exist at this interface: Insights into the evolution of viral social interactions have immediate implications for our understanding of the fundamental biology and clinical manifestation of viral diseases. However, these opportunities are currently limited because evolutionary biologists only rarely study social evolution in viruses. Here, we bridge this gap by (1) summarizing the ways in which viruses can interact socially, including consequences for social evolution and evolvability; (2) outlining some open questions raised by viruses that could challenge concepts within social evolution theory; and (3) providing some illustrative examples, data sources, and conceptual questions, for studying the natural history of social viruses.

## 1 | INTRODUCTION

Viruses offer exceptional opportunities for students of social evolution. Social interactions between viruses are pervasive, diverse, and span the spectrum of sociality. Mutually beneficial interactions underpin fundamental viral traits such as genome replication and the construction of virions, while the breakdown of these interactions opens the door to viral cheats, which are known to exploit almost all known viruses (Díaz-Muñoz et al., 2017; Leeks et al., 2021; Sanjuán, 2021).

Social interactions can drive viral population dynamics, shape viral genome evolution, and influence clinical outcomes. Some viral social interactions are so pervasive that they have already been studied for decades within virology, providing a wealth of empirical knowledge that evolutionary biologists could use. For example, collective viral transmission has been known about since the 1930s, phenotype mixing since the 1950s, and viral cheating has been studied since the 1940s, when defective interfering genomes were observed in the earliest tissue culture infections of Influenza virus (Bald & Briggs, 1937; Novick & Szilard, 1951; von Magnus, 1947). Other viral social interactions are newly discovered, revealed by recent advances within virology that are expanding the frontiers of what was thought possible for viruses. For example, recent discoveries have shown that half of bacterial *Escherichia coli* genomes contain integrated viral cheats, that plant viruses can share gene products across infected tissues, and that phages inside neighbouring bacterial cells communicate in order to coordinate their lysis timings (Box 1; Erez et al., 2017; Moura de Sousa & Rocha, 2022; Sicard et al., 2019).

The existence of these viral social traits, both new and existing, provides many opportunities for both virology and evolutionary biology. From the perspective of virology, evolutionary theory can unify otherwise disparate viral entities, provide new answers to old questions, and offer new directions for empirical research (Díaz-Muñoz et al., 2017; Domingo-Calap et al., 2019; Leeks et al., 2021, 2023; Turner & Chao, 1999; Wild et al., 2009; Wilke, 2005). From an evolutionary perspective, viruses represent a new arena for testing existing theory, offer a range of new puzzles to explore, and can both challenge and extend established bodies of theory (Borges et al., 2018; Landsberger et al., 2018; Michalakis & Blanc, 2020; Shirogane et al., 2021, 2023; Sicard et al., 2019; Skums et al., 2015).

However, these opportunities are currently held back by a lack of integration between these fields, especially since evolutionary biologists only rarely study social traits in viruses. There is a divide between empirical virologists, who study the details of virus-virus interactions, and evolutionary biologists, who study the causes and consequences of social interactions.

Here, we provide a primer for evolutionary biologists interested in studying social evolution in viruses (‘sociovirology’). We focus on theoretical and comparative questions, that can be answered without requiring virus-specific experimental techniques. We start by outlining the ways that viruses interact, illustrating some of the diversity of viral social traits. Then we explore some specific theoretical challenges and opportunities for social evolution theory that are raised by viral social interactions. Finally, we discuss how to integrate natural history into the study of social interactions in viruses, highlighting conceptual challenges and the kinds of data that can be used to tackle them.

## 2 | HOW CAN VIRUSES BE SOCIAL?

### 2.1 | What is sociality?

A social interaction occurs when a trait carried by one individual affects the fitness of a different individual (Glossary) (West et al., 2007b). Social traits have been studied for decades within evolutionary biology, resulting in a large body of theoretical and empirical work (Hamilton, 1964; West et al., 2007a). One reason for this is that social traits often lead to extraordinary phenotypes, that appear evolutionarily puzzling, and that are often successfully explained through the adoption of new theoretical frameworks. For example, the introduction of game theory to biology in the 1970s transformed the way that animal societies were studied, while developments in kin selection since the 1960s have had far-reaching impact across multiple disciplines within biology and beyond (Bourke, 2011; Crespi et al., 2014; Hamilton, 1964; Kurzban et al., 2015; Maynard Smith & Price, 1973; West et al., 2021). Understanding the evolution of social traits can also help us to explain other problems within biology. For example, theoretical frameworks developed to explain the evolution of cooperative groups have in turn successfully explained the origins of organismal complexity through major transitions in individuality (Boomsma, 2022; Bourke, 2011; Maynard Smith & Szathmary, 1995; West et al., 2015). Many of the theoretical ideas behind sociality were originally developed with animal behaviour in mind, but these frameworks have now expanded, and the prolific field of ‘sociomicrobiology’ encompasses a wide variety of microbial traits, such as multicellularity in slime moulds, group-formation in green algae, and the production of public goods in bacteria (Griffin et al., 2004; Kapsetaki & West, 2019; Strassmann et al., 2000). More recently, ideas from social evolution have started to be applied explicitly within virology, resulting in the emerging field of sociovirology (Díaz-Muñoz et al., 2017; Leeks et al., 2021; Sanjuán, 2021).

### 2.2 | Causes of viral sociality: coinfection in viruses

Viruses can interact socially whenever multiple viral genomes infect the same cells, hosts, or populations of hosts (Box 1). In an evolutionary sense, each physically distinct viral genome is usually considered to be an individual. This is because larger spatial scales, such as the virion, the virus-infected cell, or the viral population, often contain multiple genetically distinct viral genomes that can be in evolutionary conflict (Díaz-Muñoz et al., 2017; Leeks et al., 2021; Sanjuán, 2017). In this review, we follow the standard definition of treating each physically distinct viral genome as an individual, although later we discuss alternative definitions of a viral individual (Question 5). We will discuss social interactions that occur between different viral genomes from the same viral species, and between viral genomes from taxonomically distinct viruses (Glossary).

Social interactions between viruses have far-reaching consequences for viral evolution (Box 1). On the one hand, viral social interactions allow for cooperation and conflict, which drive viral population dynamics, pathogenicity, and evolution. On the other hand, viral social interactions influence the evolutionary potential for viral populations to respond to changes in environments (evolvability; Glossary). We cover the mechanistic details of viral cooperation and cheating only briefly here because recent reviews cover these topics in depth. (Díaz-Muñoz et al., 2017; Domingo-Calap et al., 2020; Leeks et al., 2021; Sanjuán, 2021). In Box 1 we illustrate some of the kinds of viral social interaction that are possible, outlining how different aspects of the viral lifecycle can depend upon social interactions (Box 1).

### 2.3 | Consequences of viral sociality: cooperation in viruses

Cooperation is a type of social interaction in which a trait carried by one individual evolves because it increases the lifetime reproductive success of another individual (West et al., 2007b). Cooperation has historically been treated as a problem in evolutionary biology because its evolution was initially difficult to explain: why help another when you could help yourself? There are two kinds of answers (West et al., 2007a).

One route to cooperation comes via direct fitness benefits. This occurs when the costs of cooperation are ultimately recouped by the individual carrying the cooperative trait (the actor) (Glossary). For example, if cooperation increases the likelihood that the actor itself receives cooperation in the future, or if cooperation allows the actor to avoid punishment or sanctions from another individual (Axelrod & Hamilton, 1981; Kiers et al., 2003). Examples of cooperation that are maintained by direct fitness benefits include the fixation of nitrogen by *Rhizobium* bacteria and the production of light by symbiotic *Aliivibrio fischeri* bacteria inside *Euprymna scolopes* bobtail squid (Kiers et al., 2011; Nyholm & McFall-Ngai, 2004).

Cooperation can also be maintained by indirect fitness benefits, via evolutionary altruism (Glossary). In altruistic cooperation, the effect of cooperation on the lifetime reproductive success on the actor is a net negative (West et al., 2007a). In these cases, kin selection can lead to altruistic cooperation being favoured, provided that the benefits of cooperation primarily go to genetic relatives of the actor (indirect fitness benefits) (Glossary) (Hamilton, 1964). Altruistic cooperation includes many extreme examples of cooperation, such as sterile worker castes in eusocial insects and the self-sacrificial production of fruiting bodies in *Dictyostelium discoideum* slime moulds or *Myxococcus xanthus* bacteria (Hughes et al., 2008; Strassmann et al., 2000; Velicer et al., 2000).

In many cases, both direct and indirect benefits can be important. In some cases, both routes to cooperation can even matter for the same trait: vampire bats preferentially share food both with relatives, and with non-relatives who have previously shared food with them, indicating that both direct fitness benefits (in the form of reciprocity) and indirect fitness benefits are important (Carter & Wilkinson, 2013; Wilkinson, 1984); cooperatively breeding birds will help at the nest for a mixture of indirect fitness benefits (gained by helping relatives to reproduce) and direct fitness benefits (for the chance of inheriting good breeding positions) (Downing et al., 2018; Griffin & West, 2003); and many host-symbiont mutualisms depend on both altruism within the symbiont population, and sanctions imposed by the host on its symbionts (Herre et al., 1999; Kiers et al., 2003; Leeks, dos Santos, et al., 2019).

In viruses, both altruism and mutual benefits can drive the evolution of cooperation. For example, many animal viruses produce molecules that suppress the release of interferon from infected cells. This is an altruistic trait, that is maintained by kin selection; it is selected for only when genetic relatedness is high, and the benefits of suppressing interferon go to relatives (Domingo-Calap et al., 2019; Leeks & West, 2019). A different kind of altruistic viral trait occurs when viruses grow at slower rates, altruistically limiting their virulence; such ‘prudent’ mutants have been selected for via kin selection in evolution experiments conducted on phages (Kerr et al., 2006; Wild et al., 2009).

In many cases, both mutual benefit and altruism can operate on the same viral trait, analogous to food-sharing in vampire bats, cooperative breeding in birds, or host-microbe mutualisms. For example, viral replicase enzymes, that replicate the viral genome, are typically shared between multiple viral genomes inside the same cell. The shared nature of the viral replicase is typically selected for when the benefits primarily go to related viral genomes, and selected against otherwise, suggesting that altruism is important (Leeks et al., 2021). At the same time, the shared nature of the viral replicase can create a positive feedback loop between the numbers of replicase templates and replicase enzymes, resulting in a direct fitness benefit being returned to genomes encoding the replicase enzyme (Andreu-Moreno et al., 2020).

In some cases, viral social interactions are mutually beneficial, but might not be cooperation in the evolutionary sense, because the trait could be maintained for reasons that are unrelated to the benefit the trait provides to another individual. Examples of such traits, where a mutual benefit exists but could be incidental to the evolution of the trait, include the production of proteins that suppress bacterial CRISPR immune systems, the increased population productivity of genetically diverse viral populations, or interactions where the presence of one viral variant influences the extent to which the immune system targets other viral variants (Borges et al., 2017; Landsberger et al., 2018; Skums et al., 2015; Vignuzzi et al., 2006). In these cases, mutual benefits are clearly important consequences of the viral trait in question. However, to test whether the mutual benefits also play a role in the evolutionary maintenance of the trait, experiments would need to test whether the trait is still favoured when the benefits do not go to other individuals that also carry the trait.

### 2.4 | Consequences of viral sociality: cheating in viruses

Both routes to cooperation open the door to cheating, and viruses appear to be especially susceptible to the evolution of cheats (Ghoul et al., 2014; Leeks et al., 2021). Cheats are a type of parasite that are favoured by natural selection because they exploit the benefits of cooperation, without paying the costs (Glossary). In comparison with the rest of the living world, cheats appear to be particularly widespread, diverse, and abundant in viruses (Leeks et al., 2021).

The most prolific known kind of viral cheats are defective interfering genomes. These are viral mutants that carry a deletion in a gene for a shared gene product, and which spread by exploiting copies of that gene product encoded by other viruses (Huang & Baltimore, 1970). Defective interfering genomes emerge de novo in almost all viruses that are grown in tissue culture, are found in natural viral infections, and can achieve orders-of-magnitude fitness advantages over cooperators (Aaskov et al., 2006; Saira et al., 2013; Shirogane et al., 2021; Vignuzzi & López, 2019). These cheats can be so effective that they are being developed as a new type of antiviral therapy; to date, defective interfering genomes have been used successfully to treat at least nine species of virus (Chaturvedi et al., 2021; Dhar & Bandyopadhyay, 2014; Johnson et al., 2021; Levi et al., 2021; Rezelj et al., 2021; Scott et al., 2011; Welch et al., 2022).

Beyond defective interfering genomes, other types of viral cheat can also be found widely (Leeks et al., 2021). This provides an opportunity to draw comparisons between different types of viral cheat, in order to explore the evolutionary forces shaping cooperation and cheating in viruses. For example, defective interfering genomes often appear rapidly and spread quickly, but typically go extinct within the timecourse of an individual infection, and are thought to only rarely transmit between hosts. This means their evolution may be ‘short-sighted’, characterized by rapid evolution, but lacking complex adaptations that may take more time to emerge (Lythgoe et al., 2017). In contrast, other viral cheats frequently transmit between hosts, and hence persist over much longer timescales; examples include virophages that exploit giant viruses, satellite viruses in plants that exploit their helper viruses, and phage satellites that integrate into bacterial genomes (Hackl et al., 2021; La Scola et al., 2008; Rocha & Bikard, 2022; Simon et al., 2004). These cheats that persist over longer timescales may be less constrained by ‘short-sighted’ evolution, and hence often display complex adaptations for exploiting cooperative viruses, such as when phage satellites reshape the viral capsid to preferentially include cheat genomes over cooperator genomes (Fillol-Salom et al., 2019; Ram et al., 2012). The ability to draw such comparisons is a key advantage to studying sociality in viruses (Leeks et al., 2021); we explore several other such comparisons later in this article.

### 2.5 | Consequences of viral sociality: evolvability in viruses

Viral sociality can also have direct consequences for the evolvability of viral populations. At the most basic level, viral coinfection allows for recombination, reassortment, and other forms of genetic exchange between viruses (Glossary) (Simon-Loriere & Holmes, 2011). At the same time, coinfection allows for the sharing of viral public goods such as replicase and capsid proteins, which means that the phenotype of one virus may depend on the genotypes of other coinfecting viruses (Box 1). One immediate consequence of this is that the tempo of natural selection can be weaker when coinfection is more common, in an analogous way to how selection can act more strongly on haploid organisms than on polyploid ones (Hunter & Fusco, 2022; Immler & Otto, 2018; Wilke, 2005; Wilke & Novella, 2003). Empirically, this means that when coinfection is rare, non-adaptive defective variants that lack key gene products are quickly selected against. However, defective variants can persist in the population provided coinfection rates are high enough to allow frequent coinfection with functional variants (phenotype masking/hiding) (Bushman & Antia, 2019; Wilke & Novella, 2003). Similarly, if viral variants share replicase enzymes, then the mutation rate of one variant may depend on the replicase enzyme encoded by a different variant.

Social interactions among viruses can also have indirect consequences for evolvability over ecological timescales, by driving viral population dynamics. For example, the invasion of viral cheats frequently leads to a collapse in the viral population, which reduces the effective population size (Leeks et al., 2021). The reduced effective population size subsequently decreases both the mutational supply and the strength of selection on beneficial alleles, which can prevent viral populations adapting to new selection pressures (Bell & Collins, 2008; Meir et al., 2020; Singhal & Turner, 2021). Feedbacks between these different processes could also be possible: smaller viral populations are likely to experience reduced rates of coinfection, which could then increase the strength of selection by reducing the phenotypic masking effects discussed in the previous paragraph.

**FIGURE 1:**
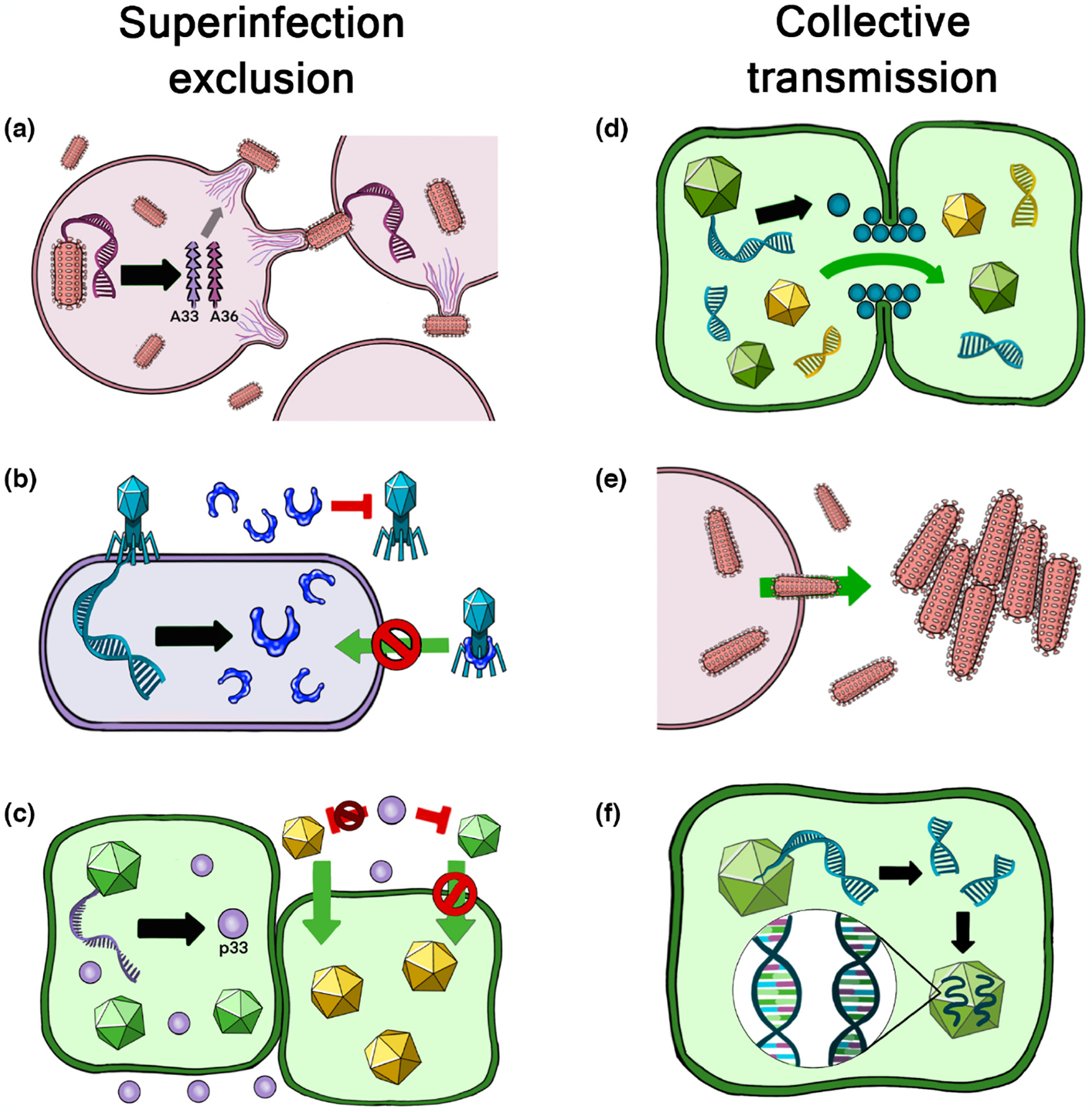
Viral traits can influence group size. We provide some illustrative examples of viral traits that influence the rate of coinfection. On the one hand, many viruses have mechanisms of superinfection exclusion, which reduce the rate of coinfection: (a) in animals, viruses such as vaccinia, viruses trigger host cells to produce surface receptors that prevent further infection by vaccinia (Doceul et al., 2010); (b) in bacteria, phages such as T4 unleash a lysozyme enyme that degrades other phage genome copies that enter the cell (Shi et al., 2020); (c) in plants, virus such as Citrus Tristeva Virus release proteins that can prevent other infections at the level of the whole host (Bergua et al., 2014). On the other hand, many viruses have mechanisms of collective infection, that increase the rate of coinfection: (d) many plant viruses release proteins that allow viral genomes and gene products to stream directly into neighbouring host cells (Sanjuán, 2018); (e) in animal viruses, virions often aggregate after leaving a host cell (Andreu-Moreno & Sanjuán, 2018); (f) in a range of viruses including HIV and measles, virions can contain multiple copies of the viral genome (Rager et al., 2002).

Social interactions can also drive viral adaptations in ways that have longer term consequences for evolvability. For example, the potential for beneficial viral social interactions can favour viruses with increased rates of coinfection, such as via collective infectious units (Leeks, Sanjuán, et al., 2019; Sanjuán, 2017). Conversely, the risks of cheating could favour viruses to decrease the rate of coinfection, such as via superinfection exclusion (Figure 1). Cheating can even drive the evolution of new forms of genome organization, that in turn allow new mechanisms of evolvability or genetic plasticity. For example, the sequential invasion of cheats can drive the evolution of segmented and multipartite viral genomes, resulting in a viral population where the genome is split across multiple physically distinct segments (Leeks et al., 2023; Nee, 1987). This new type of genome organization then allows new mechanisms of genetic exchange, such as reassortment between distinct genome segments (Simon-Loriere & Holmes, 2011), as well as phenotypically plastic ways of responding to environmental change, via expressing genome segments at different levels across different hosts (Gallet et al., 2022; Zwart & Elena, 2020).

A final possibility is that social interactions can change the constraints on viral genomes, allowing new areas of genetic space to be explored. In particular, many viral cheats emerge through deletions or loss-of-function mutations; in such cheats, the sections of their genomes that previously coded for those genes can now freely evolve new mutations without impacting the fitness of the cheat. Consequently, the genomes of cheats often contain more genetic variation than the genomes of cooperators (Aaskov et al., 2006; Gelbart et al., 2020). If recombination can subsequently occur between cheats and wild-type viruses, this variation could then be reincorporated into the wild-type viral genome. One consequence of cheating could therefore be the exploration and then incorporation of new kinds of genetic variation that would not otherwise have been available to the viral population. This mechanism is more likely to be possible in viruses with higher rates of recombination, such as some families of positive-strand RNA viruses, and less likely to be possible in viruses in which recombination is rare, such as negative-strand RNA viruses (Glossary) (Simon-Loriere & Holmes, 2011). This proposed mechanism would be analogous to the way in which gene duplications allow Eukaryotic genomes to explore new areas of sequence space (Ohno, 1970).

## 3 | OPPORTUNITIES AND CHALLENGES FOR NEW THEORY

There are clear links between established evolutionary theory and empirical examples of viral sociality. However, there is often a disconnect between the way that evolutionary biologists and virologists study viral sociality, with different researchers often starting from very different sets of evolutionary assumptions (Díaz-Muñoz et al., 2017; Domingo et al., 2012; Leeks et al., 2021; Wilke, 2005). New theory can be useful to resolve such discrepancies, by making assumptions explicit, and by generating predictions that allow us to falsify specific hypotheses. In this section, we outline five questions that provide opportunities for new evolutionary theory to resolve discrepancies between virology and evolutionary biology.

### 3.1 | Can cheating be adaptive in viruses?

When cheats spread within a viral population, they often drastically reduce the growth of that population. Typically, this will be costly for the viral population, and can even result in population extinction (Kirkwood & Bangham, 1994). However, an alternative outcome is that the accumulation of cheats can transform the viral infection from a short-lasting, acute infection, into a long-lasting, chronic one (Poirier et al., 2018; Ruiz-Gómez et al., 2021; Sun et al., 2015; Xu et al., 2017). This transformation can occur via at least two routes: firstly because viral cheats directly interfere with the growth of the wild-type virus by competing for intracellular gene products; and secondly because many viral cheats disproportionately stimulate host immune responses, resulting in a stronger immune response that subsequently suppresses the wild-type virus (Manzoni & López, 2018). Cheat-induction of immune responses appears to be a general phenomenon, and has has been well-documented among viruses infecting animals, plants, and phages (Hynes et al., 2014; Manzoni & López, 2018; Szittya et al., 2002).

The link between viral cheating and chronic infections has led a number of virologists to argue that this could provide a benefit to the viral population in the long run (Alnaji & Brooke, 2020; Poirier et al., 2018; Vignuzzi & López, 2019; Xu et al., 2017). The argument is that chronic infections could create more opportunities for between-host transmission, and hence wild-type viruses could be selected, in some cases, to evolve their genomes in ways that will increase the production of viral cheats. If viral genomes have evolved to produce cheats, this could imply that the generation of cheats is a mechanism by which the wild-type virus manipulates host immunity to induce a chronic infection. Often, the mechanistic details by which viral cheats stimulate host immunity are given as evidence that cheats have been shaped by natural selection for this purpose. Such mechanisms have now been described in some detail; in animal cells, defective viral genomes can stimulate host immunity via RNAi, apoptosis, and interferon-induction (Poirier et al., 2018; Ruiz-Gómez et al., 2021; Sun et al., 2015; Xu et al., 2017).

If cheats really do provide long-term benefits to the viral population, this would be a sharp departure from the typical consequences of cheat-cooperator interactions elsewhere in biology (Ghoul et al., 2014). If viruses have evolved to produce cheats, this would be even more surprising, as cooperators elsewhere in nature tend to evolve in exactly the opposite direction: cooperation often requires mechanisms that constrain cheating; cooperators often display costly and intricate adaptations for avoiding interacting with or being exploited by cheats; and cooperators can even be favoured to structure their genomes in ways that reduce the rate at which cheats are generated (Davies, 2010; dos Santos et al., 2018; Ghoul et al., 2014).

Two kinds of evolutionary theorizing would be particularly useful here. Firstly, theoretical models could clarify the conditions under which this ‘adaptive cheating’ argument could work; these models would need to incorporate relationships between virulence, between-host fitness, and within-host viral social interactions. For example, in terms of overall between-host transmission opportunities, can the benefits of a longer-lasting infection outweigh the costs of a reduced viral population size (Frank, 1996)? If so, why would cheating be selected as a mechanism to reduce viral growth rate, and not any other change that results in slower spread, such as a slower rate of replication (Fitzsimmons et al., 2018)? Secondly, are there alternative evolutionary explanations for why viral cheats disproportionately stimulate host immune responses (Manzoni & López, 2018)? One hypothesis that could be tested is that viral cheats are a reliable cue of a viral population reaching high densities, and hence will activate host immunity when it is most needed, whereas other cues of viral presence may risk triggering immune responses when they are not needed, such as when hosts come into contact with inactive viral material. A related hypothesis could be that viruses are typically under selection to hide from immune detection, and can achieve this via structural changes to their sequence, but that these structural changes are disrupted when cheats evolve via large deletions, and hence those cheats become detectable by the host in a way that the wild-type viral genomes are not.

### 3.2 | How do different timescales influence viral social interactions?

The evolutionary success of viruses depends on their ability to spread both within and between hosts (Read, 1994). This can result in a trade-off, whereby traits that are beneficial at one timescale are harmful at another (Lythgoe et al., 2017). For example, viruses that exploit their hosts too quickly can decrease their potential to transmit to new hosts, and viruses that adapt too closely to their current host may be less effective at infecting new hosts (Lythgoe et al., 2013).

This trade-off can also apply to the fitness consequences of social interactions between different viral entities. One area where this could be particularly relevant is in the world of satellite viruses, which are viral entities that rely on another virus to successfully infect a host (Simon et al., 2004). Some satellites are clearly cheats at the within-host level, replicating at the expense of their helper virus. Other satellites appear to be more mutualistic, sometimes increasing the replication of the helper virus (Gnanasekaran & Chakraborty, 2018). However, such satellites that are mutualistic at one timescale could be parasitic at another timescale, and vice versa. For example, a satellite that increased its helper virus’s replication within a host may end up exacerbating the symptoms of the viral infection, ultimately reducing the transmission opportunities available to the helper virus (Frank, 1996). Cooperator/cheat coevolution in viruses could thus depend upon conflicting selection pressures at the within- and between-host levels (Lythgoe et al., 2017). These evolutionary distinctions could also inform clinical treatment strategies by predicting the long-term effects of an intervention. For instance, a therapeutic viral cheat that dampened symptoms of an infection could potentially prolong that infection, causing more infections in the long run.

More broadly, these examples illustrate that viral social interactions can have different evolutionary consequences over different timescales. This raises questions about how best to define different types of viral entity, and how selection might act over each of these timescales. For example, if an entity is a cheat over one timescale but a mutualist over another, then should we expect antagonistic or mutualistic coevolution between the two entities? Does this depend on the relative importance of each timescale for adaptation? How commonly should we expect conflict between different timescales in the evolution of viral social interactions? To what extent are the relative importance of the different timescales in viral evolution influenced by host-level adaptations?

### 3.3 | How should we model the population genetics of viral coinfections?

When multiple viral genomes infect the same host cell, the phenotype of each virus depends on the genotype of all of the genomes present. There is a direct analogy here with the concept of ploidy in population genetics, which refers to the number of copies of each genome that determine the phenotype of an organism (Hartl & Clark, 2006; Wilke, 2005). This means that the degree of coinfection will have direct consequences, both for the tempo of natural selection, and on the capacity for viral populations to evolve in response to change.

We can understand some of these consequences by drawing analogies to population genetics. Within population genetics, a body of theory exists describing how ploidy influences the mode and tempo of natural selection (Gerstein & Otto, 2009). Some of these ideas have been directly applied to explain the population dynamics of viruses when coinfections occur. For example: deleterious variants can persist for longer when coinfection rates are higher, which is analogous to higher ploidy masking selection on deleterious alleles; different viral variants can coexist stably if coinfections are more productive than single infections (analogous to heterozygote advantage) (Bushman & Antia, 2019; Leeks et al., 2018; Wilke & Novella, 2003); and in meales virus, mutations for cell–cell fusion that are deleterious when combined on the same genome are beneficial when split across multiple co-transmitting genomes, in an example of viral social interactions reversing the sign of epistasis (Shirogane et al., 2023).

There are many more potential links here that have not yet been formally modelled. For example, in typical population genetics, organisms tend to have a fixed ploidy, or a ploidy that varies predictably between different states, as is common in sexually reproducing organisms (Mable & Otto, 1998). In contrast, in viruses, both the likelihood of coinfection and the number of coinfecting genomes are likely to vary from generation to generation (Wilke, 2005). What are the consequences of such variable ploidy for drawing insights from population genetics for viral coinfection? To what extent do existing analogies, such as to polyploidy, epistasis, or heterozygote advantage, break down when coinfection occurs only temporarily, or unpredictably? And do these insights only applied to coinfected cells by the same species of virus, or can we also apply these insights to cells or virions involving coinfection by different viral species (Haney et al., 2022)?

### 3.4 | What is the optimal group size for a virus?

Viruses have evolved many mechanisms that alter the rate of coinfection, hence influencing the opportunities for social interactions. For example, many viruses have evolved mechanisms of ‘superinfection exclusion’, which reduce the potential for social interactions by preventing multiple viruses infecting the same host cell (Bondy-Denomy et al., 2016; Doceul et al., 2010; Folimonova, 2012; Mavrich & Hatfull, 2019; Sims et al., 2022). At the same time, many viruses have evolved to increase the rate of coinfection, often via mechanisms of collective infection, that ensure the transmission of multiple viral genomes to the same cell or host (Sanjuán, 2017). Mechanisms of collective infection occur throughout the viral world, including in taxonomically unrelated groups of viruses; they commonly include proteins that allow viral genomes to transfer between host cells that are physically close, or physical structures that allow multiple viral genomes to travel together (Figure 1).

The fact that viral coinfection is at least partly under viral control raises the question of optimal group size. It is possible to make some broad predictions here, especially given that both superinfection exclusion and collective transmission are common, have evolved independently across taxonomically unrelated groups of viruses. One line of thought is that superinfection exclusion and collective infection are opposite sides of the same coin, with factors that favour one disfavouring the other. For example, negative viral social interactions, such as cheating, or deleterious recombination and reassortment, could select in favour of superinfection exclusion; this would be consistent with the finding that cheating selects against the evolution of collective infection in viruses (Andreu-Moreno & Sanjuán, 2020; Leeks, Sanjuán, et al., 2019). Conversely, positive social interactions, such as cellular-level Allee effects, can select for higher rates of coinfection by favouring the evolution of collective infectious units (Glossary) (Andreu-Moreno & Sanjuán, 2018; Leeks, Sanjuán, et al., 2019; Segredo-Otero & Sanjuán, 2019); such beneficial viral interactions imply a cost to infecting cells alone, and hence could select against superinfection exclusion.

However, the picture appears to be more complex than this because many viruses have both collective infection and superinfection exclusion (Sanjuán, 2017). How do we reconcile this apparent contradiction? One possibility is that collective infection and superinfection exclusion facilitate different types of social interaction (Sanjuán, 2021). For example, many mechanisms of collective transmission copackage viruses coming from the same cell (Figure 1); this will often lead to groups of genetically related viruses, that are unlikely to contain cheats, and hence could facilitate cooperative interactions between viruses (Leeks et al., 2021; West et al., 2007a). Such collective transmission could be consistent with superinfection exclusion, which could then prevent unrelated viruses infecting the same cell. In other cases, collective transmission involves genetically distinct viruses, such as when virions stick together after leaving host cells; such a mechanism is more likely to include genetically unrelated viruses and/or cheats, and hence may be more likely to facilitate mutually beneficial but non-cooperative types of viral social interaction (Andreu-Moreno & Sanjuán, 2020). These aggregating types of collective transmission often involve transmitting from host-to-host, or infecting new types of tissue, suggesting that the balance of benefit to risk of viral social interactions may depend on the stage of the viral lifecycle. In some cases, the details of viral biology suggest that more complex mechanisms might be at play. For instance, superinfection exclusion is often more effective at excluding viruses from the same species than genetically unrelated viruses (Bondy-Denomy et al., 2016; Folimonova, 2012). One reason that has been suggested for this could be if viruses are selected to maximize their rate of spread within a host tissue, which can be faster when each virus infects a new cell rather than multiple viruses infecting the same cell (Doceul et al., 2010); this mechanism would suggest a conflict between the rate of spread within a tissue via infecting new cells, and the expected success of infecting each host cell (Andreu-Moreno & Sanjuán, 2018). An alternative explanation could be that more distantly related viruses are less likely to be able to cheat the resident virus because they may be less able to exploit shared gene products. In reality, many of these processes may be at play, with potential for conflicts and feedbacks between them.

There is a need for more far-reaching theory to resolve these apparent paradoxes, that can unify how these different aspects of viral sociality influence viral group size. For example, can we predict the conditions under which the potential for positive interactions will outweigh the risks of negative interactions, and vice versa (Leeks et al., 2021; Sanjuán, 2018)? How does this balance depend on the details of viral biology, such as transmitting within tissues, or between hosts (Doceul et al., 2010)? When negative interactions occur, how does group size interact with other mechanisms of resistance to cheating that occur in viruses (DePolo et al., 1987; Horiuchi, 1983; Meir et al., 2020)? To what extent are there ecological and evolutionary feedbacks between the evolution of group size and the evolution of social interactions (Brännström et al., 2011; Moreno-Fenoll et al., 2017; Peña & Nöldeke, 2018; Sanchez & Gore, 2013)? Are there formal analogies to be drawn between group size in viruses and similar ideas from elsewhere in evolutionary biology (Boomsma, 2009; Bourke, 1999; Hamilton, 1971; Lack, 1947; Maynard Smith & Szathmary, 1995; Peña & Nöldeke, 2018)?

### 3.5 | What is a viral organism?

We and others have defined each physical viral genome as an individual in the evolutionary sense because this is the largest unit at which we expect minimal evolutionary conflict between distinct entities (Díaz-Muñoz et al., 2017; Leeks et al., 2021; Sanjuán, 2021). However, viruses frequently interact with one another at larger spatial scales, such as when they coinfect the same cells or hosts (Box 1). Could it ever be useful to define a viral individual at one of these larger spatial scales?

The reason this might be useful would be if viral traits exist whose evolution can only be explained at these larger spatial scales. There is an analogy here with the on-going effort to define an organism within evolutionary biology, as well as with the related field that studies major transitions in individuality (Bourke, 2011; Díaz-Muñoz et al., 2016; Maynard Smith & Szathmary, 1995; Okasha, 2009; Patten et al., 2023; Queller & Strassmann, 2009; West et al., 2015). In these fields, researchers typically consider how organisms are adapted to their environments, while recognizing that adaptation occurs via natural selection causing changes in allele frequencies (Ågren & Patten, 2022; Davies et al., 2012; Gardner, 2009). This logical extension works because genes within an organism typically share a single route for onwards transmission, and so they have aligned fitness interests. Hence, organisms are defined by a combination of minimal evolutionary conflict together with a high degree of cooperation between constituent units (Gardner & Grafen, 2009; Queller & Strassmann, 2009). Consequently, we can think about adaptation at the level of whole genomes that consist of multiple genes, multicellular organisms that consist of multiple cells, and even eusocial superorganisms that themselves consist of numerous multicellular organisms (Boomsma, 2022; Bourke, 2011; Maynard Smith & Szathmary, 1995).

To what extent could the evolutionary concept of the organism carry over to groups of viruses? In most cases, it seems that the analogy of the organism breaks down because organisms are defined by minimal conflict, but there is substantial evidence for conflict within viral populations, as evidenced by the abundance of cheating (Leeks et al., 2021). At the same time, there are examples of viral cooperation occurring at larger spatial scales, such as when animal viruses cooperatively block the release of interferon from infected cells (Box 1), or when plant viruses share public goods across infected tissues (Domingo-Calap et al., 2019; Sicard et al., 2019). In an evolutionary sense, the presence of both conflict and cooperation means that viral infections are more analogous to societies than to organisms (Queller & Strassmann, 2009).

Viral organismality is an area where careful theory would be useful, linking the evolutionary idea of organismality to viral biology. How common is cooperation between viruses at spatial scales that extend beyond the infected cell, and can we predict when this will occur (Box 1) (Domingo-Calap et al., 2019; Sicard et al., 2019)? To what extent does within-host evolution create conflict between viruses sharing a host (Leeks et al., 2021; Lythgoe et al., 2017)? Can viruses evolve more complex ‘organism-like’ adaptations, such as policing, as is seen in within genomes, multicellular organisms, and eusocial insects (Ågren et al., 2019; Frank, 1995; Ratnieks et al., 2006)? At the other end of the scale, does the presence of selfish genetic elements, such as homing endonucleases in T4 phages, generate sufficient conflict to undermine individual viral genomes themselves as cohesive evolutionary units (Edgell et al., 2010; Gardner & Úbeda, 2017; Patten et al., 2023)? Finally, how do concepts of evolutionary individuality apply when viral genomes are themselves split into different segments (segmented viruses), and especially when those segments can transmit independently (multipartite viruses) (Holmes, 2009; Leeks et al., 2023; Lucía-Sanz & Manrubia, 2017; Michalakis & Blanc, 2020)?

## 4 | THE NATURAL HISTORY OF VIRUSES

The most productive areas of evolutionary biology are defined by the interplay between natural history, controlled experiments, and theoretical modelling. Typically, observations of organisms in their natural environment reveal puzzling traits, for which an evolutionary explanation is required. Evolutionary biologists then conduct theoretical studies, to test the plausibility of different hypotheses, and to make quantitative, testable predictions that allow hypotheses to be falsified and refined (Davies et al., 2012; West et al., 2021). In the most successful subfields of evolutionary biology, such as sex allocation, this process has resulted in an extraordinarily close quantitative fit between theoretical and empirical results, to a degree that is rarely seen within the life sciences (West, 2009).

Such a combination of approaches will be especially useful for studying social evolution in viruses. From a basic science perspective, it is only possible to understand how natural selection has shaped viruses if we understand the natural environments within which viruses evolve. For example, experimental evolution studies can indicate what kinds of processes could drive viral adaptation, but we can only test whether these processes have actually driven viral adaptation if we know the selective conditions that viruses face in their natural environments. Bridging this divide is also essential from an applied perspective, especially regarding the rapidly expanding clinical interest in exploiting viral social interactions for predicting clinical symptoms and for treating viral infections (Chaturvedi et al., 2021; Dhar & Bandyopadhyay, 2014; Felt et al., 2021; Johnson et al., 2021; Levi et al., 2021; Rezelj et al., 2021; Scott et al., 2011; Welch et al., 2022).

But what does natural history mean for viruses? Unlike many other living organisms, it is not intuitively obvious how to observe and study viruses in their natural habitat – most viruses cannot be seen even under a light microscope. We suggest that one fruitful way to explore the natural history of viruses is through genomic datasets of viruses in their natural environments. Many such datasets have already been collated and made publicly available for other purposes, such as for environmental microbiology or clinical surveillance (Table 1). Alternatively, it is increasingly feasible for researchers to generate their own viral metagenomic datasets, using relatively unspecialised techniques, that can then be analysed using freely available computational tools and infrastructure (Roux et al., 2019, 2021).

In this section, we provide some examples of how publicly available datasets have already been used to answer key questions about social evolution in viruses, and we discuss some of the conceptual challenges for using comparative methods to study viruses.

### 4.1 | Integrating natural history into sociovirology

A key question in sociovirology is the extent to which viruses coinfect cells. Or, from an evolutionary perspective, which social partners are interacting? A number of studies have used metagenomic datasets to provide coinfection estimates in different natural systems (Díaz-Muñoz, 2017; Munson-McGee et al., 2018; Roux et al., 2014). Depending on the type of metagenomic data, coinfection can be quantified explicitly at the level of single cells or hosts, or can be inferred indirectly from the ratio of viral particles to host cells. In the future, the advent of meta-transcriptomic and conformation sequencing methods could move beyond just identifying the social partners, to pinpoint the functional and physical interactions occurring among viruses.

Another direct use is to detect cheats within viral genome sequencing datasets. As outlined above, cheating variants are frequently detected in lab experimentation, often involving large genomic deletions. Thus, there are opportunities to use genomic data directly to examine to what extent viral cheats exist outside the lab, as well the clinical and evolutionary consequences of cheats. Saira et al. (2013) verified that influenza viral cheats are indeed present in clinical infections, opening the door for future studies that infer cheat prevalence, cheat-cooperator population dynamics, and the consequences of cheats for clinical outcomes (Felt et al., 2021; Leeks, 2021; Martin et al., 2019; Saira et al., 2013; Vasilijevic et al., 2017). As sequencing technology advances rapidly, so too do the opportunities for investigating viral cheats in nature. For example, long-read sequencing has the potential to capture the entire sequence of a viral cheat, which can be particularly useful when viral cheats are formed from multiple deletions or more complex rearrangements (Jaworski & Routh, 2017; Routh, 2018). Similarly, cost-effective and user-friendly sequencing technologies now exist for specifically this purpose, such as ClickSeq, which can precisely identify the sequence of cheats, as well as quantifying their relative abundance (Jaworski & Routh, 2017; Routh & Johnson, 2014).

Evolutionary biologists can also glean insights into viral social evolution by examining *bacterial* genomic data sets. Many bacterial genomes contain satellite phage cheats integrated inside them (so-called ‘sit-and-wait’ cheats) (Leeks et al., 2021; Rocha & Bikard, 2022). These satellites typically lie dormant until their bacterium is infected by their host phage strain, upon which they activate and exploit the incoming phage, such as by encoding genes that restructure the phage capsid to contain satellite genomes instead of wild-type phage (Christie & Dokland, 2012; Novick et al., 2010; Ram et al., 2012). Many integrated satellite cheats have now been well-studied in the laboratory, including P4-like elements, which are found in almost 50% of *E. coli* genomes, Staphylococcal pathogenicity islands (SaPIs), which can reduce the production of wild-type phage by 500-fold, and ICEs in Vibrio cholerae, whose coevolutionary dynamics can influence the epidemiology of cholera outbreaks (Frígols et al., 2015; McKitterick & Seed, 2018; Moura de Sousa & Rocha, 2022). Recent work has exploited the fact that these cheats can be discovered in bacterial genomes, combined with mechanistic knowledge of how they work, to rapidly expand the repertoire of known integrated satellites in bacterial genomes (Chevallereau & Westra, 2022; Millman et al., 2022; Moura de Sousa et al., 2023). Detailed studies have then unpicked the evolutionary dynamics shaping some of these cheats, identifying genes conserved between phylogenetically distant satellites, revealing that phylogenetically similar satellite cheats can be found in distant bacterial genomes, and finding evidence for long-term coevolutionary dynamics among different gene modules required for exploiting cooperative phages (Moura de Sousa & Rocha, 2022). There are analogies here to how experiments and population genomics data have been combined to explore cheats elsewhere in nature (Belcher et al., 2022; de Oliveira et al., 2019; Noh et al., 2018; Ostrowski et al., 2015). More broadly, the widespread presence of viral cheats that are integrated within bacterial genomes means that now is an excellent time to investigate the natural history of these kinds of integrated viral cheats (Bobay et al., 2014; Christie & Dokland, 2012; Novick et al., 2010; Penadés & Christie, 2015; Rocha & Bikard, 2022).

In some cases, more specific principles regarding viral social dynamics can be inspired by and addressed using genomic data, integrated with laboratory experimentation. For instance, a key question is the extent to which social population dynamics that are observed under laboratory conditions also play out in natural viral infections. Xue et al., 2016 used clinical data to discover a new potential type of beneficial interaction between Influenza variants that coexisted in sequencing datasets from laboratory-passaged cultures (Xue et al., 2016). They confirmed experimentally that these influenza variants complemented each other in cell entry and exit, performing better together than either one individually; subsequent theoretical work showed that this synergistic benefit can maintain viral diversity in a way that is directly analagous to the concept of heterozygote advantage in population genetics (Leeks et al., 2018). Xue et al. (2018) then sought these variants in clinical isolates to explore if this phenomenon occurred in natural infections, and used further experimentation to confirm it did not within the human hosts they looked at. This work showed that there is the potential for a mutually beneficial interaction between the variants, but that the conditions required for this interaction to take place were not met in the natural infections examined (Xue et al., 2018). This set of studies demonstrates the potential for interplay between natural history, experimentation, and theory that is possible by making use of public data sources to study social evolution in viruses.

### 4.2 | Comparative methods in sociovirology

One particularly powerful way to use natural history datasets is by using phylogenetic comparative methods. These methods allow researchers to test the causal influence of factors across a broad range of species over evolutionary timescales, while accounting for the fact that closely related species are often phenotypically similar (Felsenstein, 1985; Harvey & Pagel, 1991; Revell & Harmon, 2022). Comparative methods occupy a central place in modern evolutionary biology, and have resolved key questions in topics including sex allocation, major transitions theory, and the evolution of cooperation (Davies et al., 2012; Frank, 2022; Harvey & Pagel, 1991; Hughes et al., 2008; Lukas & Clutton-Brock, 2013; West, 2009).

Within virology, it is more common to use phylogenetic methods to infer other details of viral population biology. For example, methods rooted in birth-death models and coalescent theory have been used to date the origins of viral outbreaks, to infer the number of independent introductions of viral lineages during epidemics, and to estimate the relative growth rates of different viral variants during epidemics (du Plessis et al., 2021; Grenfell et al., 2004; Volz et al., 2021). When phylogenetic comparative methods have been used to study questions of viral adaptation, this has typically been confined to specific groups or lineages of viruses. For example, Frígols et al. (2015) found that in staphylococcal phages, regions of the genome conferring resistance to exploitation by cheats remained highly variable even among closely related phages, suggesting that cheat-cooperator coevolutionary dynamics play out in natural phage populations (Frígols et al., 2015).

Applying phylogenetic comparative methods to test hypotheses about social evolution in viruses could be highly fruitful, and will underpin any attempt to make broad claims about social evolution and the natural history of viruses. However, these studies are currently held back by a combination of factors, both technical and conceptual. Current technical limitations include: limited phenotypic data on most viruses; unresolved viral phylogenies; the likelihood of multiple independent viral origins; and limited viral sampling, such that current datasets likely represent a tiny and biased fraction of the Earth’s true viral diversity (Krupovic et al., 2019; Lefkowitz et al., 2018; Prangishvili et al., 2017; Roux et al., 2019). Fortunately, the current era of viral discovery is leading to rapid technical and computational advances, both for the discovery of new viruses, and for predicting phenotypes of interest from genomic datasets (Edgar et al., 2022; Gregory et al., 2019; Roux et al., 2021). Consequently, more data is becoming available that will allow formal phylogenetic comparative studies in viruses in the near future.

However, from the evolutionary side, there is also a need for theoretical advances in phylogenetic comparative methods themselves. For example, how do we formally test comparative hypotheses when there are multiple independent origins of viruses, resulting in multiple independent phylogenies (Koonin et al., 2022)? How do we deal with phylogenetic trees that are defined by multiple horizontal transmission events, including between phylogenetically distant groups of viruses? If viral evolution is modular and rapid, and relatively unconstrained by recent evolutionary history, then do we need to control for phylogeny in the same way as elsewhere in biology (Felsenstein, 1985)? To what extent do the problems that we have pointed out for virology have parallels elsewhere in evolutionary biology?

## 5 | CONCLUSION

The social lives of viruses present a wealth of possibilities for both evolutionary biology and virology. Capitalizing on these opportunities will require tools and expertise from evolutionary biology, together with knowledge of viral social interactions. Here, we have outlined some outstanding problems that could be immediately tackled by evolutionary biologists, using existing frameworks within social evolution theory, population genetics, and comparative biology. Solving these problems will open the door to fundamental advances in evolutionary theory, coupled with empirical findings that have direct clinical relevance.

## Figures and Tables

**TABLE 1:** Publicly available viral genomic data.

Resource	Type	Type of virus	Environment	Host	Source data	Reference
IMG/VR	Genomic	Prokaryotic	All	Bacteria, Archaea	Community submitted	Roux et al. (2021)
GISAID	Genomic	Eukaryotic	Clinical and Environmental	Human and animal	Genomic surveillance	Shu and McCauley (2017)
EpiFlu	Genomic	Influenza		Human and animal	Genomic surveillance	Shu and McCauley (2017)
ICTV	Taxonomy	All	All	All	Expert opinion	Lefkowitz et al. (2018)
Serratus	RdRp	RNA viruses	All	All	NCBI SRA	Edgar et al. (2022)
NCBI SRA	Genomic	All	All	All	Community submitted	Leinonen, Sugawara, et al., (2011)
NCBI RefSeq	Genomic	All	All	All	Community submitted	Brister et al. (2015)
ENA	Genomic	All	All	All	Community submitted	Leinonen, Akhtar, et al. (2011)
COG-UK	Genomic	SARS-CoV-2	Clinical	Human	Genomic surveillance	COG-UK (2020)
Viral zone	General Ref	All	All	All	Literature, expert opinion	Hulo et al. (2011)

## Data Availability

There is no new data to report.

## BOX 1 Social interactions in viruses across spatial scales.

At each stage of the viral lifecycle, there is the opportunity for social interactions between viral genomes.

Whenever multiple viral genomes coinfect a host cell, there is the possibility that they share gene products. Two gene products that are commonly shared are the replicase enzyme (a), that replicates the viral genome, and capsid proteins (b), which construct the viral capsid that transports viral progeny to new host cells. Shared gene products can be considered public goods in the evolutionary sense because encoding them is usually costly for individual viral genomes, but provides a shared benefit (Díaz-Muñoz et al., 2017; Leeks et al., 2021).

Viruses can also interact when they infect neighbouring cells. One example is when viruses interfere with cellular signalling, such as when mammalian viruses encode molecules that block host cells’ interferon-based immunity (c) (Domingo-Calap et al., 2019). Another example is when plant viruses produce gene products that can be shared across different host cells (d) (Sicard et al., 2019).

For viruses that infect single-celled organisms such as Bacteria or Archaea, each infected cell is also a host, and interactions can occur between viruses infecting different hosts. Some of these interactions have direct analogues to sociality in viruses of multicellular hosts. For example, many phages encode ‘anti-CRISPR’ proteins in order to infect their CRISPR-carrying bacterial hosts, which is analogous to interferon-blocking in mammalian viruses (e) (Borges et al., 2017; Domingo-Calap et al., 2019). Anti-CRISPR proteins tend to be only partially effective, meaning that these phages rely on collective benefits from multiple or sequential infections to successfully infect a host (Borges et al., 2018; Landsberger et al., 2018). In other cases, viruses of single-celled organisms have evolved ways of interacting that have not yet been seen elsewhere in viruses, such as when viruses of *Bacillus* bacteria use quorum-sensing ‘arbitrium’ molecules to coordinate the time at which they burst their bacterial hosts (f) (Erez et al., 2017)

## GLOSSARY

Social trait: A trait that influences the fitness of another individual.
Cooperation: A trait that provides a benefit to the lifetime reproductive sucess of a recipient individual, and is favoured by natural selection because of this benefit.
Allee effects: A positive relationship between population size and the mean fitness of individuals.
Altruism: A type of cooperation in which the cooperative trait is costly to the individual that bears it, but beneficial to the recipient, where cost and benefit are defined in terms of lifetime reproductive success.
Cheat: An individual that does not contribute (or contributes less) to a cooperative trait, but gains the benefits of other individuals’ cooperation.
Kin selection: The process by which trats can be favoured due to their effects on individuals that are genetically related to the individual carrying the trait.
Reassortment: A process in which genome segments are swapped between individuals.
Recombination: A process in which sections of genome are mixed between different genome segments.
Ploidy: The number of genome copies that contribute to an organism’s phenotype.
Coinfection: The presence of multiple viral genomes inside the same cell, host, or group of hosts.
Evolvability: The potential for a viral population to respond evolutionarily to change.
Sociovirology: A field studying social evolution in viruses.
Defective viral genomes: A non-standard viral genome that is unable to successfully infect cells on its own.
Defective interfering viral genomes: A subtype of defective viral genome, which can successfully infect cells when coinfecting with a cooperative wild-type genome, and which consequently reduces the fitness of coinfecting wild-type viral genomes. Defective interfering genomes are evolutionary cheats.
Polyploid virions: Virions containing multiple copies of the same viral genetic material.
Segmented viruses: Viruses whose genetic information is split across multiple physically distinct strands of genetic material, in which each strand is transmitted within the same virion.
Multipartite viruses: Viruses whose genetic information is split across multiple physically distinct strands of genetic material, in which each strand is transmitted within a different virion.
Satellite virus: A viral entity that relies on the presence of another ‘helper virus’. Satellites generally share no sequence homology with their helper viruses, and can be mutualists, parasites, or commensals.
Viral species: New viral species are defined as monophyletic groups that are distinguishable from others in their genus, as determined by the International Committee on Taxonomy of Viruses (ICTV) (Lefkowitz et al., 2018).
